# Expanding the Phenotype of Homozygous *KCNMA1* Mutations; Dyskinesia, Epilepsy, Intellectual Disability, Cerebellar and Corticospinal Tract Atrophy

**DOI:** 10.4274/balkanmedj.2017.0986

**Published:** 2018-07-24

**Authors:** Gözde Yeşil, Ayşe Aralaşmak, Enes Akyüz, Dilara İçağasıoğlu, Türkan Uygur Şahin, Yavuz Bayram

**Affiliations:** 1 Department of Medical Genetics, Bezmialem Vakıf University School of Medicine, İstanbul, Turkey; 2 Department of Radiology, Bezmialem Vakıf University School of Medicine, İstanbul, Turkey; 3 Department of Child Disease and Health, Bezmialem Vakıf University School of Medicine, İstanbul, Turkey; 4 Department of Child Neurology, Bezmialem Vakıf University School of Medicine, İstanbul, Turkey; 5 Mol. & Human Gene/Lupski Lab, Baylor College of Medicine, Texas, USA

**Keywords:** Cerebellar atrophy, dyskinesia, epilepsy, KCNMA1, spinal tract atrophy

## Abstract

**Background:**

The *KCNMA1* gene encodes the α-subunit of the large conductance, voltage, and calcium-sensitive potassium channel (BK channels) that plays a critical role in neuronal excitability. Heterozygous mutations in *KCNMA1* were first illustrated in a large family with generalized epilepsy and paroxysmal nonkinesigenic dyskinesia. Recent research has established homozygous *KCNMA1* mutations accountable for the phenotype of cerebellar atrophy, developmental delay, and seizures.

**Case Report:**

Here, we report the case of a patient with a novel homozygous truncating mutation in *KCNMA1* (p.Arg458Ter) presenting with both the loss- and gain-of-function phenotype with paroxysmal dyskinesia, epilepsy, intellectual delay, and corticospinal–cerebellar tract atrophy.

**Conclusion:**

This report extends the *KNCMA1* mutation phenotype with a patient who carries a novel frameshift variant, presenting with both the gain- and loss-of-function phenotypes along with spinal tract involvement as a novel characteristic.

The *KCNMA1* gene encodes the a-subunit of the large conductance, voltage, and calcium-sensitive potassium channel (BK channels), which is also activated by the concentration of cytosolic Mg^2+^ and is known to be predominantly expressed in the amygdala, caudate nucleus, cerebral cortex, hippocampus, hypothalamus, spinal cord, and Purkinje cells in the cerebellum (1,2). Initially, the *KCNMA1* mutations were illustrated in a large family with generalized epilepsy and paroxysmal nonkinesigenic dyskinesia (3). A recent study established a correlation of the homozygous *KCNMA1* mutation with cerebellar ataxia, developmental delay, and seizures. In addition, both the gain- and loss-of-function have been proposed as the underlying molecular mechanism in this channelopathy resulting in increased excitability (4). Here, we report the case of a patient with a novel homozygous truncating mutation in *KCNMA1* (p.Arg458Ter) presenting with both the loss- and gain-of-function phenotype with paroxysmal dyskinesia, epilepsy, intellectual delay, and corticospinal–cerebellar tract atrophy.

## CASE PRESENTATION

**Figure 1:**
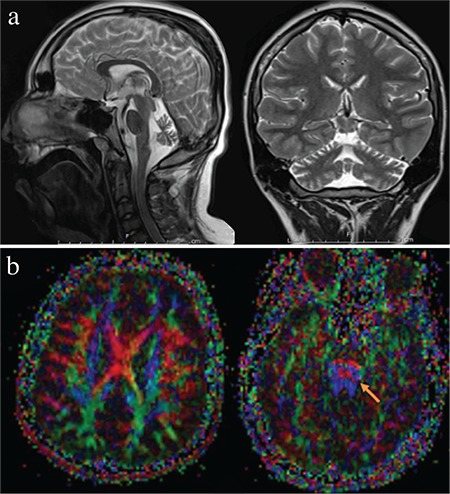
Sagittal and coronal brain magnetic resonance images of a 14-year-old boy revealed cerebellar vermian volume loss with normal pons and spinal canal (a). Diffusion tensor imaging images displayed thinning of the tegmental extending through corticospinal tracts (b).

**Figure 2:**
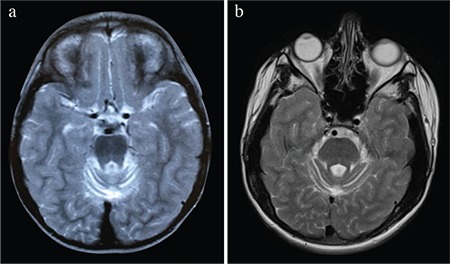
Compared to previous magnetic resonance imaging, the atrophy of the cerebellum progressed; (a) performed when he was of 3 years and (b) performed 12 years after the initial magnetic resonance imaging.

A 15-year and 11-month-old male patient was referred to our genetics unit at the age of 15 years. He was born at term to a third-degree consanguineous healthy parents with a healthy birth weight (3250 g), height (53 cm), and occipitofrontal circumference (34 cm). There was a prolonged labour, and the APGAR score was 7-8. His motor milestones were delayed, and he never walked alone. In addition, he had a social smile and could talk approximately 10 simple words. His seizures, although mostly absent, started at the age of 18 months and were well-controlled by valproic acid. Meanwhile, he also experienced clonic and generalized tonic–clonic (GTCS) and atonic seizures and had spasticity predominant in the lower extremities with no pathological reflexes. While electroencephalography revealed generalized spike-wave activities, electromyography and metabolic tests were normal. Furthermore, the brain magnetic resonance imaging (MRI) performed at the age of 3 years revealed moderate atrophy with prominent folia in the upper parts of the supratentorial cerebellar vermian region. Moreover, symmetric T2 hyperintensities were observed at the retroatrial periventricular deep white matter. Diffusion tensor imaging images obtained at the age 14 years revealed the involvement of tegmental to corticospinal atrophy (Figure 1). Besides, the atrophy of the cerebellum had progressed compared to previous MRI studies (Figure 2). The patient’s last examination determined contractures on the large joints, dyskinetic tremor, and dystonia. Of note, this study was reported per the tenets of the Declaration of Helsinki and was approved by the institutional review board and ethical committee of our university. We obtained written informed consent from the patient.

The exome sequencing revealed a homozygous nonsense change in the *KCNMA1* gene NM_001161352.1:c.1372[C>T];[C>T] NP_001154824.1:p.[(Arg458*)];[(Arg458*)]. The variant was not observed in any publicly available database (e.g., EXAC, EVS, and 1000 genomes) or in our internal database. In addition, we identified another variant, rs60734921, in the *CACNAH1* gene, which has been described in a study as a risk factor for generalized idiopathic epilepsy (5). While the population frequency of the variant in the *CACNAH1* gene was 0.0012/39 according to the EXAC, it was classified as a variant of unknown significance in the dbSNP database (Table 2).

## DISCUSSION

Large-conductance calcium-sensitive BK channels are one of the potassium channels that hyperpolarize the neurons and are encoded by *KCNMA1* (6,7). Reportedly, mutations in *KCNMA1* have been identified in clinical cases of epilepsy and paroxysmal nonkinesic dyskinesia (3,8). In addition, a study functionally investigated the *D434G* mutation of *KCNMA1* by the patch clamp method and was found to be associated with the gain of function (3). Some studies have suggested that the gain of function at BK channels resulted in the faster and rapid repolarization of the action potential in the syndrome mechanism, accounting for an increase in the excitability of the brain (3,9). Moreover, Sausbier et al. (2,10) reported that *KCNMA1-/-* mice exhibited abnormal eye-blink reflex, abnormal locomotion, and abnormal motor coordination. Thus, either gain- or loss-of-function mutations might result in the disease phenotype. Furthermore, both the gain- and loss-of-function phenotype can be observed in other channelopathies such as *KCNA2*, *GRIN1*, and *DEAF1* gene mutations.

Recently, Tabarki et al. (4) reported a different phenotype of the same gene. In their study, the siblings were homozygous for a frameshift variant in *KCNMA1* and had tractable myoclonic seizures starting around the age 1, which later evolved into tonic and GTCS type. In addition, they had a severe developmental delay, but no dyskinesia, and their brain MRI revealed cerebellar atrophy that was not a feature of previously reported heterozygous mutations (4). Table 1 summarizes the clinical and characteristic features of patients with *KCNMA1* mutations. Unlike previous reports, our case had corticospinal and tegmental tract involvement besides cerebellar atrophy, which could be attributed to the possible progressive course of the disease attributive to the advanced age of our patient. In addition, our patient had dyskinesia and dystonic movements, which were not known for biallelic mutations. Reportedly, the variant found in the *CACNA1H* gene could also contribute the proband’s phenotype; however, the variant is a known single nucleotide polymorphism that was considered a risk factor for generalized epilepsy but not the dyskinesia phenotype (5).

In conclusion, this report presents a unique case of a patient who manifested both phenotypes of the gain- and loss-of-function mutations of *KCNMA1* (dyskinesia, epilepsy, and cerebellar atrophy) and had tegmental and spinal tract atrophy that has not been reported to date. Thus, electrophysiological analyses and expression studies are warranted to gain an insight into functional consequences of biallelic mutations of the *KCNMA1* gene. Overall, this study highlights the importance of using exome sequencing techniques for expanding the disease phenotypes to reveal the disease pathogenesis.

## Figures and Tables

**Table 1:**
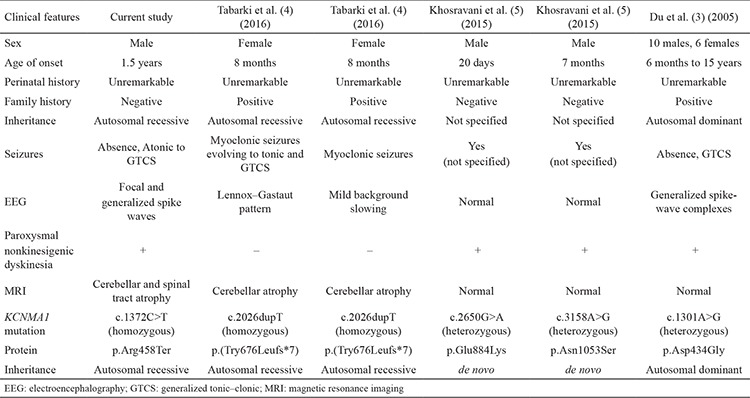
The clinical and characteristic phenotype of patients with the *KCNMA1* gene mutation

**Table 2:**
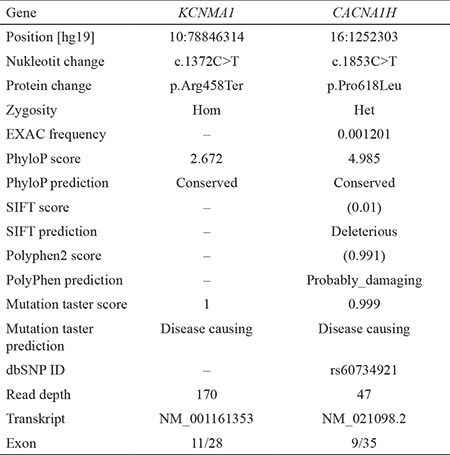
Annotations, frequency, and bioinformatic prediction scores of variants in select candidate genes
